# Rural Road Extraction in Xiong’an New Area of China Based on the RC-MSFNet Network Model

**DOI:** 10.3390/s24206672

**Published:** 2024-10-16

**Authors:** Nanjie Yang, Weimeng Di, Qingyu Wang, Wansi Liu, Teng Feng, Xiaomin Tian

**Affiliations:** 1 School of Remote Sensing and Information Engineering, North China Institute of Aerospace Engineering, Langfang 065000, China; yangnj2022@163.com (N.Y.); d2918951794@126.com (W.D.); w972913536@163.com (Q.W.); liu_ws0915@126.com (W.L.); teng_f2024@163.com (T.F.); 2 Hebei Collaborative Innovation Center for Aerospace Remote Sensing Information Processing and Application, Langfang 065000, China

**Keywords:** rural roads, RC-MSFNet, residual neural network, connectivity attention mechanism, atrous convolution, Gaofen-2

## Abstract

High-resolution remote sensing imagery, reaching meter or sub-meter levels, provides essential data for extracting and identifying road information. However, rural roads are often narrow, elongated, and have blurred boundaries, with textures that resemble surrounding environments such as construction sites, vegetation, and farmland. These features often lead to incomplete extraction and low extraction accuracy of rural roads. To address these challenges, this study introduces the RC-MSFNet model, based on the U-Net architecture, to enhance rural road extraction performance. The RC-MSFNet model mitigates the vanishing gradient problem in deep networks by incorporating residual neural networks in the downsampling stage. In the upsampling stage, a connectivity attention mechanism is added after dual convolution layers to improve the model’s ability to capture road completeness and connectivity. Additionally, the bottleneck section replaces the traditional dual convolution layers with a multi-scale fusion atrous convolution module to capture features at various scales. The study focuses on rural roads in the Xiong’an New Area, China, using high-resolution imagery from China’s Gaofen-2 satellite to construct the XARoads rural road dataset. Roads were extracted from the XARoads dataset and DeepGlobe public dataset using the RC-MSFNet model and compared with some models such as U-Net, FCN, SegNet, DeeplabV3+, R-Net, and RC-Net. Experimental results showed that: (1) The proposed method achieved precision (P), intersection over union (IOU), and completeness (COM) scores of 0.8350, 0.6523, and 0.7489, respectively, for rural road extraction in Xiong’an New Area, representing precision improvements of 3.8%, 6.78%, 7.85%, 2.14%, 0.58%, and 2.53% over U-Net, FCN, SegNet, DeeplabV3+, R-Net, and RC-Net. (2) The method excelled at extracting narrow roads and muddy roads with unclear boundaries, with fewer instances of omission or false extraction, demonstrating advantages in complex rural terrain and areas with indistinct road boundaries. Accurate rural road extraction can provide valuable reference data for urban development and planning in the Xiong’an New Area.

## 1. Introduction

Roads are vital arteries that connect the economic lifelines of different regions and their accurate extraction is crucial for the development of human society. Road information has been widely used in applications such as map updating, urban planning, and emergency response [1]. The construction of the Xiong’an New Area is one of China’s major national development strategies, with plans to establish a modern, intelligent, and eco-friendly city. As the Xiong’an New Area is still in development, rural roads currently make up a significant portion of the road network. Accurate extraction of these rural roads is essential for driving local economic growth. With advancements in remote sensing technology, the resolution of remote sensing images has reached meter or sub-meter levels, making the automatic extraction of roads from high-resolution remote sensing images [2,3] a hot topic in applied research. Although high-resolution remote sensing images provide rich spatial information, rural roads are often narrow and obscured by buildings and trees and their textures can easily be confused with surrounding environments such as construction sites, vegetation, and farmland. Therefore, the accurate extraction of rural roads remains a challenge.

Over the past decades, the development of road extraction methods has evolved through a series of transformations, from mathematical morphology-based road detection to shallow machine learning and deep learning, gradually moving towards minimizing human intervention. Unsalan et al. [4] proposed a method that combines mathematical morphology operations to extract road networks, preserving narrow road regions while removing almost all small paths and noise clusters. Fang et al. [5] introduced a road extraction method for remote sensing images based on adaptive morphology, which can effectively extract roads from complex background information in remote sensing images with high accuracy. The development and refinement of machine learning have improved the accuracy of road extraction and enhanced the efficiency of large-scale data extraction. Wang et al. [6] proposed a road extraction method that combines K-means clustering with SVM (Support Vector Machine), achieving a road extraction accuracy of 92.15%, a redundancy error of 0.19%, and a missed error of 5.31%. Zhu et al. [7] developed a road extraction method based on a hybrid model of SVM and FCM (Fuzzy C-Means) using the MAP-MRF (Maximum a Posteriori–Markov Random Field) framework to train the sampler and obtain model parameters, integrating the SVM model with a fuzzy comprehensive evaluation model. To extract road surfaces, Sharma et al. [8] employed SVM and Artificial Neural Networks (ANN), noting that the ANN, being an extensive neural network, requires significant processing time and more extended training periods for large datasets [9].

In recent years, the advancement of deep learning has significantly propelled the progress of road extraction, with an increasing number of studies focusing on extracting roads from high-resolution remote sensing images using deep learning methods. This is primarily due to CNN’s (convolutional neural network) ability to extract features automatically in deep learning. CNNs can learn road features from training samples and recognize roads by combining various features. Commonly applied CNN-based models include VGG [10] (Visual Geometry Group) and FCN [11] (Fully Convolutional Networks). VGG scans every pixel in the image to identify roads, ensuring that even narrow rural roads are not missed, thus achieving fine-grained results. However, VGG relies on a small region around each pixel to determine whether it is part of a road, making it difficult to define the optimal region size—too small reduces accuracy, while too large results in computational redundancy. Additionally, the identified roads often contain more noise, leading to discontinuities. FCN uses downsampling to obtain smaller-sized feature maps, which are then upsampled to the original image size to produce the final extraction results. This upsampling–downsampling structure effectively reduces interference from complex backgrounds but tends to overlook the details of narrow roads.

The U-Net network structure consists of an encoder that captures semantic information and a decoder with a symmetrical structure to the encoder, which performs the downsampling and upsampling processes, respectively. Each downsampling stage is connected to the corresponding upsampling stage via skip connections. In recent years, this network has been widely used in road extraction from remote sensing images. However, the original network has certain limitations under specific conditions, prompting many current studies to focus on improving the U-Net network to enhance road extraction capabilities. Zhang et al. [12] combined residual blocks with the U-Net decoder to accelerate network convergence, achieving end-to-end image road segmentation. Xu et al. [13] preprocessed remote sensing data using digital filtering and then integrated the results of two U-Net networks with deep residuals to achieve semantic segmentation of roads. Buslaev et al. [14] improved the encoder part of the U-Net network by using a ResNet-34 network pre-trained on the ImageNet dataset, enhancing road segmentation performance in remote sensing. Yang et al. [15] proposed a U-Net-based network that integrates residual learning with dense connection blocks to enhance road extraction. Incorporating dense residual blocks improves the network’s ability to leverage multi-layered features, leading to a significant increase in road segmentation accuracy. There are various methods based on DeepLab. Chen et al. [16] proposed the DeepLabV2 network, which incorporates the ASPP module into the network structure, enabling multi-scale feature extraction and significantly improving image segmentation accuracy. Later, Chen et al. [17] introduced DeepLabV3+, which integrates multi-scale features extracted from ASPP with low-level feature maps, allowing the network to capture richer feature information for more accurate extraction. Xia et al. [18] utilized ResNet as the backbone for DeepLab, achieving good results on GF-2 images. These methods do not fully consider the specific challenges of rural road extraction, and the smoothness of the predicted road boundaries still needs improvement.

In summary, most current research methods have not fully considered the geometric topology structure of rural roads as a whole, lacking sufficient utilization of contextual information [19]. At the same time, they have not considered the problem of incomplete road extraction caused by the narrow and elongated rural roads and the texture being easily confused with the surrounding environment, such as construction sites, vegetation, and farmland. This study proposes a road extraction model based on RC-MSFNet to address these issues. We applied the RC-MSFNet model to extract rural roads from high-resolution remote sensing images of China’s GF-2 satellite and the Xiong’an New Area in Hebei Province. We explored its effectiveness in extracting rural roads from high-resolution images.

## 2. Study Area and Research Data

### 2.1. Overview of the Study Area

**Figure 1:**
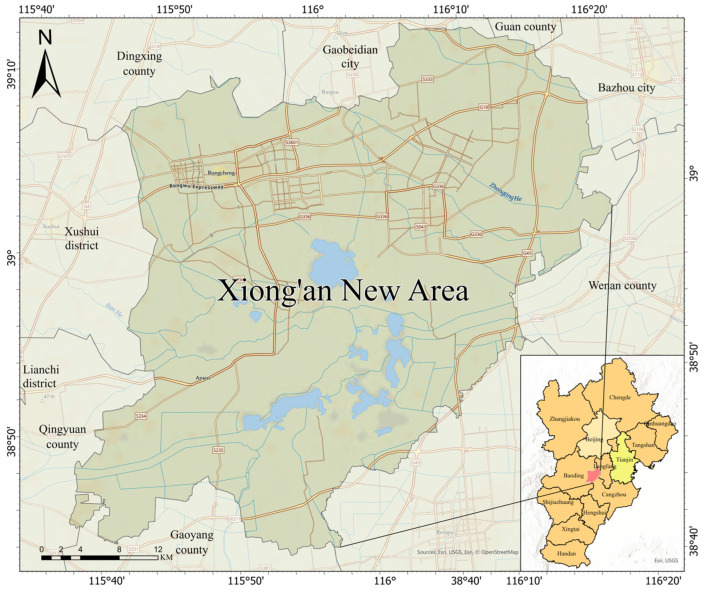
Study Area Location Map.

This study focuses on the Xiong’an New Area in Hebei Province, China, as the primary research area (Figure 1). Established as a new national area in 2017, Xiong’an is under the jurisdiction of Hebei Province and represents a significant strategic initiative by the Chinese government. The primary goals for Xiong’an include alleviating Beijing’s non-capital functions, promoting coordinated development within the Beijing–Tianjin–Hebei region, and achieving balanced regional economic growth. Strategically located in central Hebei Province, at the heart of the Beijing–Tianjin–Baoding triangle, Xiong’an holds significant strategic importance. Currently, Xiong’an New Area is predominantly rural and undergoing rapid development. The extraction and optimization of rural roads are crucial for this development, as they not only enhance transportation efficiency but also contribute to the effective allocation of resources, thereby driving rapid economic growth in the region.

### 2.2. Data Source and Preprocessing

The GF-2 satellite, successfully launched on 19 August 2014, is a domestically developed civilian optical remote sensing satellite with a spatial resolution superior to 1 m. It is equipped with two high-resolution cameras: a 1-m panchromatic camera and a 4-m multispectral camera. The satellite features sub-meter spatial resolution and high positioning accuracy. The GF-2 panchromatic data capture imagery within a spectral range of 450–900 nm, while the multispectral data consist of four bands with spectral ranges of 450–520 nm (blue), 520–590 nm (green), 630–690 nm (red), and 770–890 nm (near-infrared).

This study uses GF-2 satellite remote sensing imagery of the Xiong’an New Area, captured on 19 January 2023, as the data source. The data preprocessing was performed in ENVI 5.3, following these steps.

(1) Radiometric Calibration

For multispectral and panchromatic data, the digital number (DN) values of GF-2 imagery are converted to top-of-atmosphere (TOA) radiance using the given spectral response functions. This step translates the imagery’s digital values into physical radiative quantities, ensuring that the data accurately reflect the true optical information.

(2) Atmospheric Correction

For the multispectral data, the FLAASH algorithm, based on the MODTRAN4 model, is employed to perform atmospheric correction on the GF-2 imagery. This process aims to eliminate atmospheric influences, such as scattering and absorption of light in the atmosphere, making the imagery more representative of the true surface reflectance and enhancing the data’s authenticity and usability.

(3) Orthorectification

For both multispectral and panchromatic data, orthorectification is based on a digital elevation model (DEM). This step corrects geometric distortions caused by terrain variations and the satellite’s viewing angle, ensuring that the features within the imagery accurately correspond to their real geographic locations.

(4) Image Fusion

The processed GF-2 multispectral and panchromatic imagery, which has undergone radiometric calibration, atmospheric correction, and orthorectification, is fused. The resulting fused image retains the multispectral data’s spectral information while achieving the panchromatic data’s high spatial resolution, yielding an image with a spatial resolution of 1 m. This fusion is suitable for detailed analysis and applications.

### 2.3. XARoads Dataset Preparation

**Figure 2:**
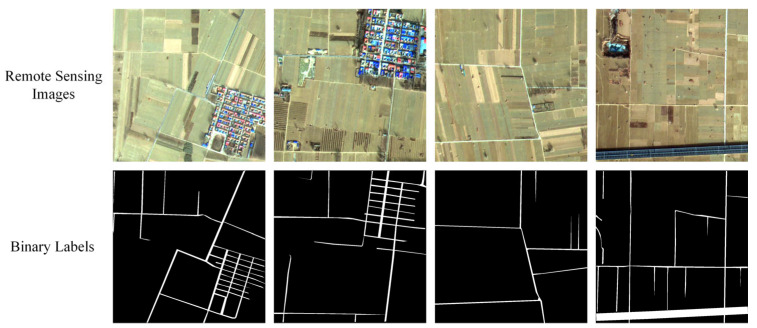
Partial Training Samples of XARoads Dataset.

To meet the input requirements for the experimental model, preprocessed remote sensing images were cropped into 1024 × 1024 pixel tiles, totaling 887 images and covering an area of 521.04 km^2^. These images primarily encompass rural roads, buildings, urban residential areas, vegetation, and highways. Manual annotation of road features was conducted using the Labelme tool, resulting in 887 road label images of the same dimensions, with white representing roads and black denoting the background. To enhance dataset diversity, augmentation techniques were applied, including image rotation, horizontal flipping, random brightness adjustments, color or contrast modifications, and random flipping. This process produced a comprehensive XARoads dataset of 1024 × 1024 pixel images, comprising 5821 original images and 5821 corresponding labels (Figure 2). The dataset was divided into training and testing sets in an 8:2 ratio [20].

### 2.4. DeepGlobe Dataset

**Figure 3:**
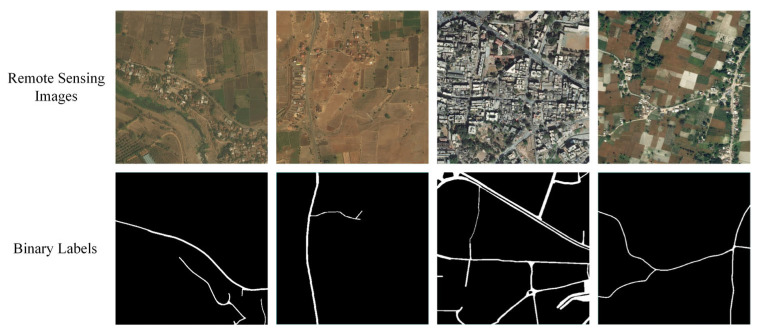
Partial Training Samples of DeepGlobe Dataset.

Demir et al. [21] introduced the DeepGlobe Satellite Road Extraction Dataset, designed to automatically extract road and street networks. The dataset consists of 6226 RGB satellite images, each measuring 1024 × 1024 pixels with a spatial resolution of 50 cm per pixel. The images were collected by Digital Globe satellites, covering diverse scenes such as urban and rural areas in Thailand, India, and Indonesia. The dataset was split into training and test sets for the experiments with an 8:2 ratio. Some of the original images and their corresponding binary labels are shown in Figure 3.

## 3. Rural Road Extraction Method

### 3.1. RC-MSFNet Network Structure

**Figure 4:**
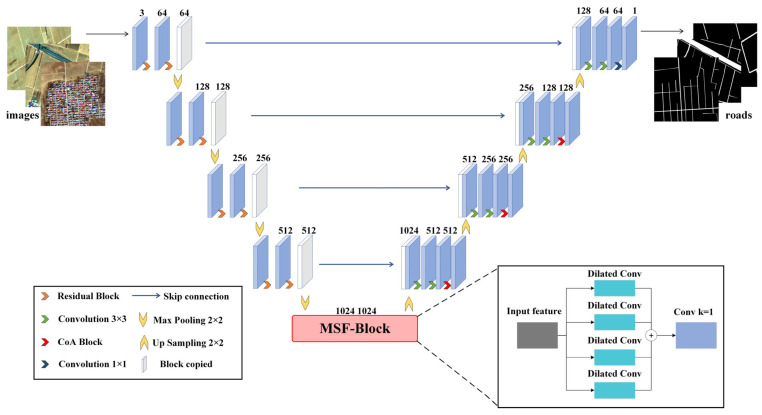
RC-MSFNet Network Model Structure.

The RC-MSFNet network structure (Figure 4) consists of three main components: the downsampling block (encoder), the bottleneck block, and the upsampling block (decoder). The RC-MSFNet model differs from the U-Net model in several key aspects: (1) it incorporates residual neural networks (ResNet) at each downsampling stage; (2) it replaces the dual-layer convolutions in the bottleneck block with multi-scale feature fusion (MSF) dilated convolution modules to capture features at different scales; (3) it integrates Channel-wise Attention (CoA) modules at each upsampling stage.

The downsampling block processes the remote sensing image data using ResNet-based dual-layer convolutional blocks, which enhances feature extraction efficiency. The downsampling process reduces the spatial dimensions of feature maps through max-pooling layers, while ResNet helps retain important information and address the vanishing gradient problem. The bottleneck block utilizes the MSF module, which employs convolutional kernels of various scales to increase the model’s ability to perceive features of different sizes. This module aids in capturing global contextual information in the image and significantly improves the handling of complex scenes, enhancing the model’s generalization capability in challenging environments. In the upsampling block, transposed convolution layers are used to incrementally restore the spatial resolution of feature maps. Following transposed convolution, CoA modules dynamically adjust channel weights to enhance the representation of essential features. Finally, a 1 × 1 convolutional layer maps the features to the final output channels. By integrating these components, the model better understands both the local and global context, effectively addressing the misclassification and omission of rural road textures that might be confused with surrounding environments such as buildings, vegetation, and farmland.

#### 3.1.1. Downsampling Block Structure

**Figure 5:**
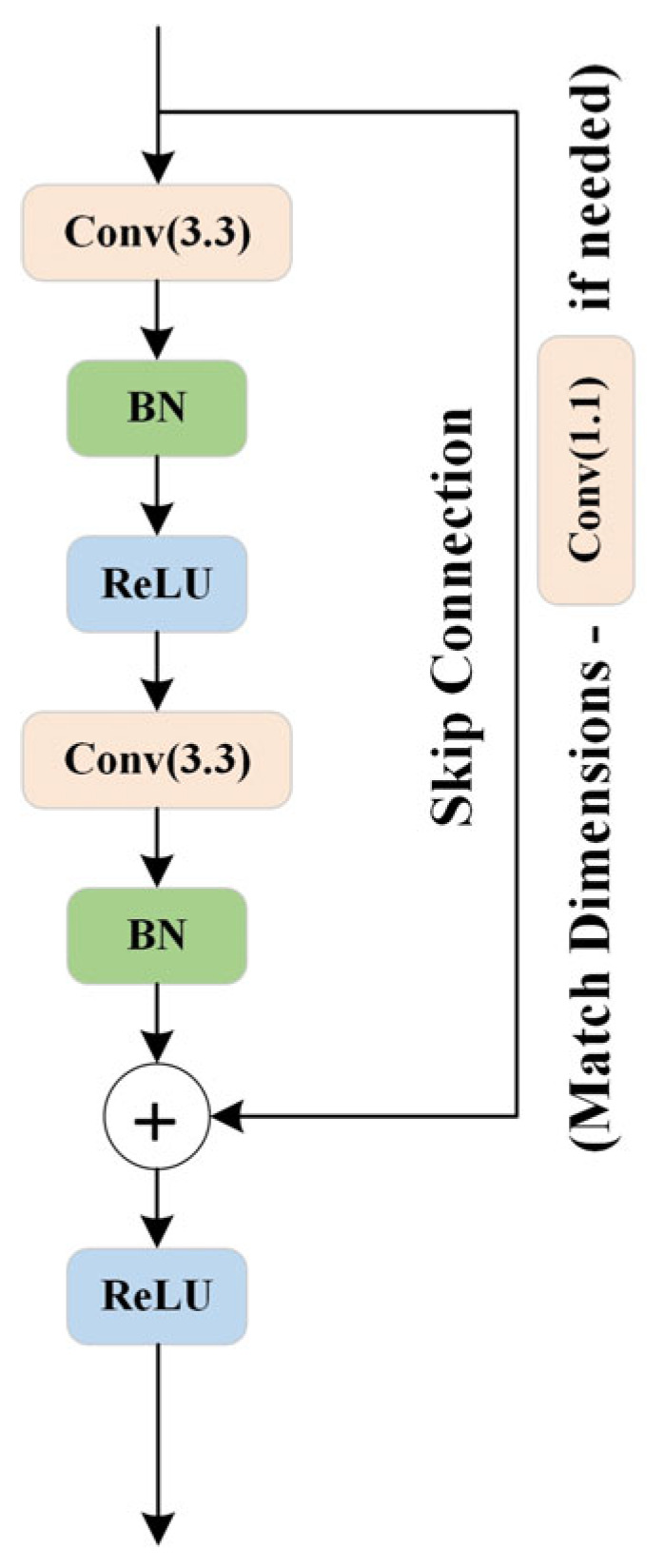
Residual Neural Network Structure Diagram.

The downsampling block’s ResNet [22] (Figure 5) is composed of two convolutional layers, each followed by batch normalization (BN) and a Rectified Linear Unit (ReLU) activation function. Both convolutional layers use 3 × 3 convolutional kernels to maintain sensitivity to spatial features. The first convolutional layer is responsible for learning the representations of the input features, while the second layer helps further abstract these features. This combination allows ResNet to adapt to varying levels of feature representations. A key feature of ResNet is its introduction of residual connections, which bypass the convolutional layers and directly pass the input features to the output of the convolutional layers. This design enables the network to learn residuals, i.e., the difference between the input and the desired output, which helps mitigate the gradient vanishing problem, particularly in deep networks. Detailed information on the ResNet structure used in the downsampling block of the RC-MSFNet model is provided in Table 1.

Additionally, to ensure that the number of input and output channels is consistent or to match dimensions when the stride is not 1, ResNet uses an optional 1 × 1 convolutional kernel for dimensionality matching [23]. An identity mapping is employed if the number of input and output channels is the same and the stride is 1. The final output of ResNet is activated using ReLU and passed on to the next network layer. This design helps achieve more stable gradient propagation in deep neural networks, thereby enhancing the effectiveness of the network’s training.

#### 3.1.2. Bottleneck Block Structure

**Figure 6:**
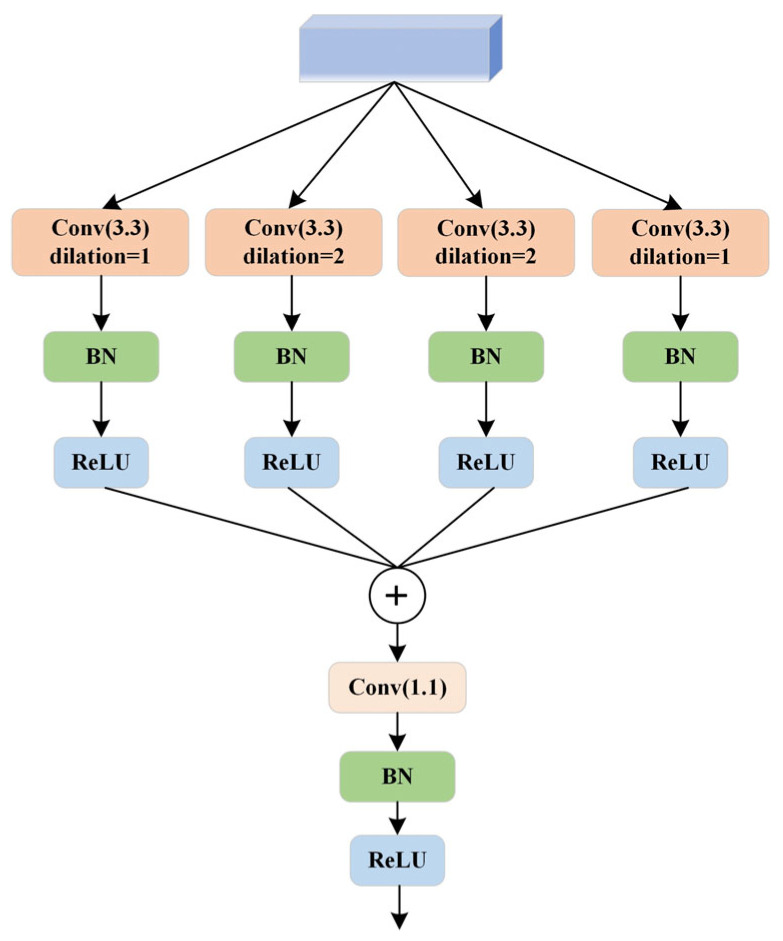
Multi-scale Fusion Atrous Convolution Module.

**Figure 7:**
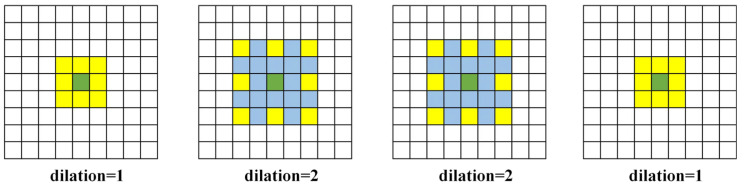
Different Levels of Atrous Convolution.

The bottleneck block features an MSF module, as illustrated in Figure 6. Dilated convolution [24] is a specialized convolution operation within convolutional neural networks. Unlike traditional convolution operations, where the convolutional kernel slides over the input feature map with a fixed stride to compute the output feature map, dilated convolution introduces holes (or dilation) into the convolutional kernel. This modification expands the receptive field of each pixel in the output feature map without increasing the number of parameters or computational load [25]. The MSF module aims to capture contextual information at different scales by using convolutional kernels with varying dilation rates, thereby enhancing the performance of neural networks in tasks such as semantic segmentation. The module is structured as a list of parallel convolutional branches, each employing dilated convolutions with different dilation rates [26]. Figure 7 shows dilated convolution kernels with dilation rates of 1, 2, 2, and 1, where the central green pixel represents the target pixel and the yellow pixels are involved in the convolution operation. Each branch consists of a sequence including a 3 × 3 convolution, batch normalization, and a ReLU activation function designed to capture features at different dilation rates. Additionally, a 1 × 1 convolutional layer fuses the outputs from all branches along the channel dimension. Detailed information about the MSF module is provided in Table 2.

#### 3.1.3. Upsampling Block Structure

The CoA module is a crucial component in convolutional neural networks. It is designed to enhance key features by adaptively adjusting channel weights. This module helps mitigate the negative impact of cluttered backgrounds—such as vegetation, farmland, and buildings—on road extraction.

**Figure 8:**
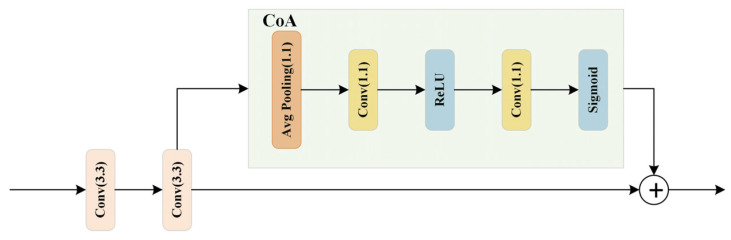
Framework Diagram of a CoA Module.

As illustrated in Figure 8, the CoA module is connected after the upsampling operation through transposed convolution layers. The process begins with a global average pooling of the input features, resulting in a global feature vector with a channel dimension of 1. This is followed by a sequence of two 1 × 1 convolutional layers: the first reduces the number of channels. In contrast, the second restores the number of output channels, learning the channel attention coefficients. The channel attention coefficients are normalized using a Sigmoid activation function, constraining the values between 0 and 1. The CoA module plays a significant role in enhancing road extraction accuracy and connectivity through the following mechanisms:

(1) The CoA module integrates a channel attention mechanism [27,28] that allows the model to focus more on channels relevant to road features while ignoring those that are not. This selective emphasis helps in enhancing the precision of road extraction.

(2) Global average pooling allows the CoA module to capture global information about the input features rather than being restricted to local regions. This global perspective helps the model better understand the overall distribution of roads and reduces the influence of local background clutter.

(3) Channel attention coefficients are computed through convolutional operations followed by a Sigmoid activation function. These coefficients dynamically adjust the weight of each channel, allowing the model to prioritize road-related features. As a result, the network becomes more adept at connecting roads accurately [29,30].

Detailed information about the upsampling block structure is provided in Table 3.

### 3.2. Experimental Design

#### 3.2.1. Parameter Settings

The RC-MSFNet network model was implemented using the PyTorch 2.0.0 deep learning framework on a Windows 10 operating system. The training was conducted on an NVIDIA GeForce RTX 4060 Ti GPU (Taipei City, Taiwan). The batch size was set to 16, with the number of worker threads configured to 2. The model’s initial learning rate was set to 0.0001.

#### 3.2.2. Comparative Experiment Design

To optimize the RC-MSFNet model parameters, we conducted experiments by adjusting the dilation rates of the atrous convolutions in the MSF and the reduction factors of the channel numbers in the CoA at various scales. Additionally, to validate the superiority of the RC-MSFNet model in rural road extraction from high-resolution remote sensing images, we conducted comparative experiments using the models listed in Table 4 against the RC-MSFNet model. The experimental data are all from the XARoads dataset created in this study, and to verify the applicability and scalability of the RC-MSFNet network, experiments will be conducted using the DeepGlobe dataset, with all experimental settings consistent with those of the RC-MSFNet model.

#### 3.2.3. Loss Function

The road extraction model typically employs the binary cross entropy (BCE) loss [31] function for training. Its calculation is expressed by Equation (1).

(1) $$L_{BCE}=-\sum_{i=1}^{N}\left(T_{i}\log\left(P_{i}\right)+\left(1-T_{i}\right)\log\left(1-P_{i}\right)\right)$$

In the equation, $P_{i}$ is used to predict the value; $T_{i}$ is for label values; $P_{i}\in \;$[0,1]; N is the total number of pixels in a sample; i is any pixel among them.

BCE loss is widely used for binary classification problems and is particularly effective for pixel-level binary classification tasks like road extraction. It optimizes by comparing the difference between the model’s predicted probabilities and the actual labels, computing the loss for each pixel.

Additionally, Dice loss is used as an alternative loss function [31]. Dice loss measures the overlap between the model’s predictions and the actual labels, with heightened sensitivity to boundary pixels during gradient descent. This helps the model better learn spatial information about the objects. Its calculation is expressed in Formula (2).

(2) $$L_{Dice}=1-\frac{2\times \sum_{i=1}^{N}P_{i}\times T_{i}}{\sum_{i=1}^{N}P_{i}+\sum_{i=1}^{N}T_{i}}$$

The final loss function is the sum of the BCE loss function and the Dice loss function [31], as expressed in Formula (3).

(3) $$L_{loss}=L_{BCE}+L_{Dice}$$

#### 3.2.4. Evaluation Indicators

To evaluate the road extraction performance of the proposed network model, four metrics are used: Precision (P), F1 Score (F1), Intersection over Union (IOU), and Completeness (COM) [32] (see Formulas (4)–(7)). P measures the model’s accuracy in predicting road areas, with higher values indicating better performance. Given extensive background regions in the images, F1 Score and IOU are typically used for comprehensive evaluation. A higher F1 Score indicates better overall classification performance, while IOU assesses the overlap between predicted and actual road areas, with higher values indicating a closer match. COM measures the integrity or connectivity of the extracted roads, with higher values suggesting fewer omissions of actual road regions.

(4) $$P=\frac{TP}{TP+FP}$$

(5) $$F1=\frac{2\times P\times \frac{TP}{TP+FN}}{P+\frac{TP}{TP+FN}}$$

(6) $$IOU=\frac{TP}{FN+TP+FP}$$

(7) $$COM=\frac{TP}{TP+FN}$$

In these formulas, TP denotes the number of true positives, where the prediction and the label are both positive; TN represents the number of true negatives, where both prediction and label are negative; FP indicates the number of false positives, where the prediction is positive but the label is negative; and FN refers to the number of false negatives, where the prediction is negative but the label is positive.

## 4. Experiment Results and Discussion

### 4.1. RC-MSFNet Model Parameter Optimization

Several experiments were conducted to validate the appropriateness of the selected parameters in this study by modifying relevant parameters in the RC-MSFNet model. These experiments were performed using the same XARoads dataset and test set. The results of these experiments are summarized in Table 5.

Table 5 shows the results of the RC-MSFNet model for rural road extraction under different parameter settings. The parameters d in the MSF module and *r* in the CoA module are adjustable within the RC-MSFNet model. The *d* parameter determines the dilation rate of the dilated convolution, directly impacting the receptive field of the model. This helps the model capture feature information at various scales. Proper dilation settings ensure that the model can both capture the broad appearance of wide roads and recognize the finer details of narrow roads. The appropriate setting of *r* can significantly enhance the model’s ability to focus on critical features, allowing the model to concentrate more effectively on essential feature regions while reducing unnecessary feature redundancy, thereby maintaining high recognition capabilities even in complex backgrounds.

As seen in Table 5, different combinations of MSF and CoA parameters affect the model’s performance to varying degrees. Adjusting these parameters ensures the model’s superiority in extracting rural roads from high-resolution remote sensing imagery. After comparing the optimization results of all network parameters, the best comprehensive performance in rural road extraction was achieved with the parameter set in Group C, where *d* was set to 1, 2, 2, 1, and *r* was set to 32. This indicates that this method can maintain high accuracy, recognition rate, and precise target localization in rural road extraction. In contrast, other parameter settings exhibited some degree of performance discrepancy or instability in these metrics.

### 4.2. Accuracy of Rural Road Extraction Using Different Deep Learning Models on the XARoads Dataset

**Figure 9:**
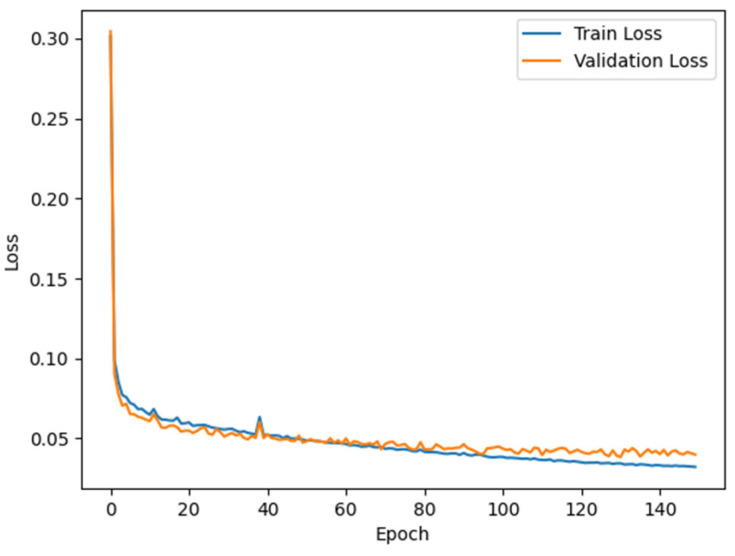
Training and Validation Loss During Model Training.

The RC-MSFNet model and U-Net, FCN, SegNet, DeeplabV3+, R-Net, and RC-Net models were trained using the XARoads dataset developed in this study. The performance of these models in rural road extraction was evaluated on the test set, and metrics such as P, F1, IOU, and COM were calculated. The results are presented in Table 6, and the loss variation during the training of the RC-MSFNet model is illustrated in Figure 9.

As shown in Table 6, the RC-MSFNet model outperforms the classic U-Net, FCN, SegNet, and DeeplabV3+ models. Compared to U-Net, the RC-MSFNet model achieves a 3.8% increase in P, a 4.82% increase in F1, a 6.33% increase in IOU, and a 5.59% increase in COM. Compared to the FCN model, the improvements are even more significant, with P increasing by 6.78%, F1 by 10.63%, IOU by 13.33%, and COM by 13.29%. Compared to the SegNet model, P increases by 7.85%, F1 by 10.76%, IOU by 13.49%, and COM by 12.80%. Similarly, against the DeeplabV3+ model, P improves by 2.14%, F1 by 9.15%, IOU by 11.61%, and COM by 13.75%.

Furthermore, RC-MSFNet also surpasses R-Net and RC-Net across all metrics. The R-Net model, which incorporates ResNet to address gradient vanishing in deep networks, shows improvements over U-Net with a 3.22% increase in P, a 1.78% increase in F1, and a 2.29% increase in IOU, but only a 0.71% improvement in COM, indicating limitations in capturing road completeness. The RC-Net model, which integrates ResNet and CoA, enhances the ability to recognize and reconstruct fragmented or partially obscured rural roads, resulting in a 2.4% improvement in COM, a 0.53% increase in F1, and a 0.69% increase in IOU compared to R-Net, though P decreases by 1.95%. The RC-MSFNet model replaces the dual convolution layers in the bottleneck region with the MSF module, which uses multi-scale dilated convolutions to expand the receptive field and capture features at varying scales to enhance rural road extraction further. This results in P improving by 2.53%, F1 by 2.51%, IOU by 3.35%, and COM by 2.48% over RC-Net, with all metrics reaching their highest values.

Figure 9 illustrates the loss variations for the RC-MSFNet model over 150 epochs on the training and validation sets. It shows that the training and validation losses decrease steadily, stabilizing the validation loss at a certain point and indicating regular model training.

### 4.3. Rural Road Extraction Effect of Different Deep Learning Models on the XARoads Dataset

**Figure 10:**
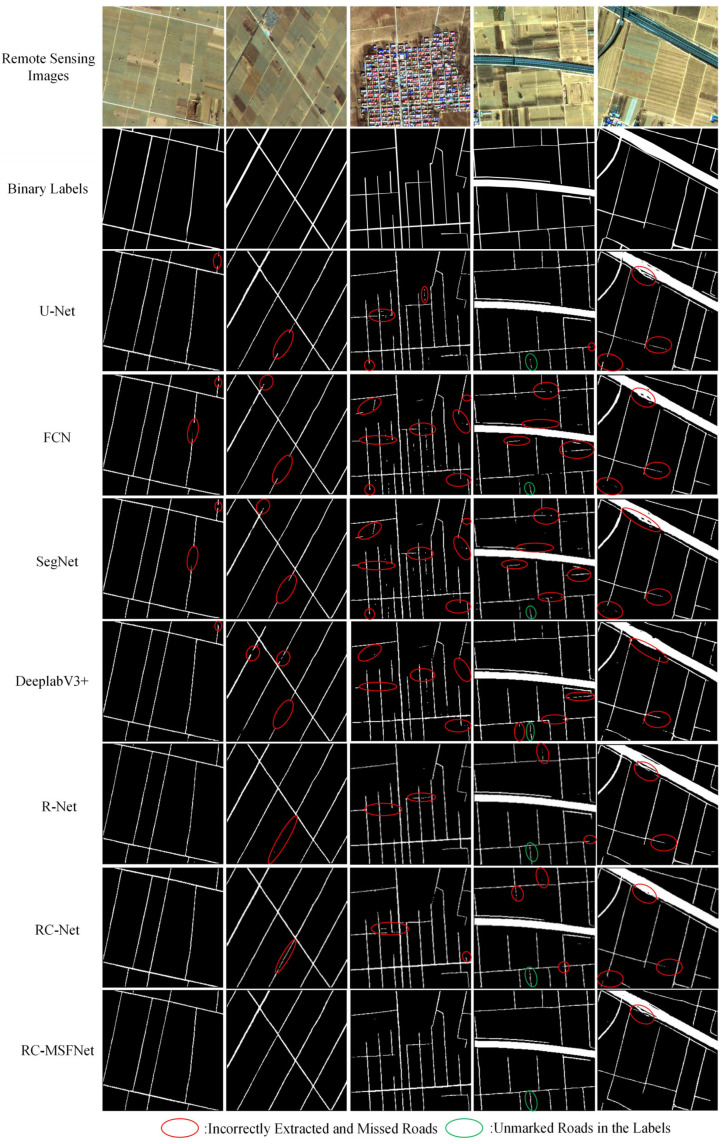
Extraction Results of Rural Roads Using Different Models on the XARoads Dataset.

Figure 10 shows the rural road extraction results of the XARoads dataset of RC-MSFNet, U-Net, FCN, SegNet, DeeplabV3+, R-Net, and RC-Net models.

As shown in Figure 10, the U-Net, FCN, SegNet, DeeplabV3+, R-Net, and RC-Net models exhibit obvious problems with incomplete road extraction, especially in narrow rural roads, poorly defined muddy roads, and shaded or unclear road boundaries. Specifically, the U-Net network performs well in road extraction, but there are also some problems. The U-Net network extracts some areas that are not roads by mistake and misses some actual roads, making some road edges unclear. The FCN network makes significant noise when extracting narrow roads and roads without clear boundaries, resulting in unclear edges on some roads. The SegNet network road extraction results could be bad, especially at the junction of roads and other ground objects; misdivision and missing division are prominent, resulting in unclear road extraction boundaries. The DeeplabV3+ model has a good extraction effect on trunk roads, but it is fuzzy on the edge of narrow roads. Compared with previous models, R-Net and RC-Net have improved performance in extracting narrow roads; however, they still missed key details, especially in areas where muddy roads were not clearly defined.

In contrast, the RC-MSFNet model performs well in road extraction by utilizing the synergistic effect of multi-scale feature fusion and the channel attention mechanism. It significantly reduces the extraction of errors and omissions and outperforms other models in extracting narrow roads and roads with unclear boundaries. The RC-MSFNet model can effectively extract road information even from unlabeled parts, demonstrating its outstanding ability to handle complex road structures.

### 4.4. Accuracy of Rural Road Extraction Using Different Deep Learning Models on the DeepGlobe Dataset

Table 7 shows the road extraction accuracy metrics of the RC-MSFNet model and U-Net, FCN, SegNet, DeeplabV3+, R-Net, and RC-Net models on the DeepGlobe dataset.

According to Table 7, the RC-MSFNet model achieved good performance on the DeepGlobe dataset (P = 0.8266, F1 = 0.7821, IOU = 0.6380, COM = 0.7422), and its road extraction metrics were superior to the U-Net, FCN, SegNet, DeeplabV3+, R-Net, and RC-Net models. Compared with the basic U-Net model, P increased by 3.59%, F1 by 2.13%, IOU by 2.83%, and COM by 0.83%. This indicates that our research method applies to the XARoads dataset and achieves high road extraction metrics on the publicly available dataset DeepGlobe.

### 4.5. Rural Road Extraction Effect of Different Deep Learning Models on the DeepGlobe Dataset

**Figure 11:**
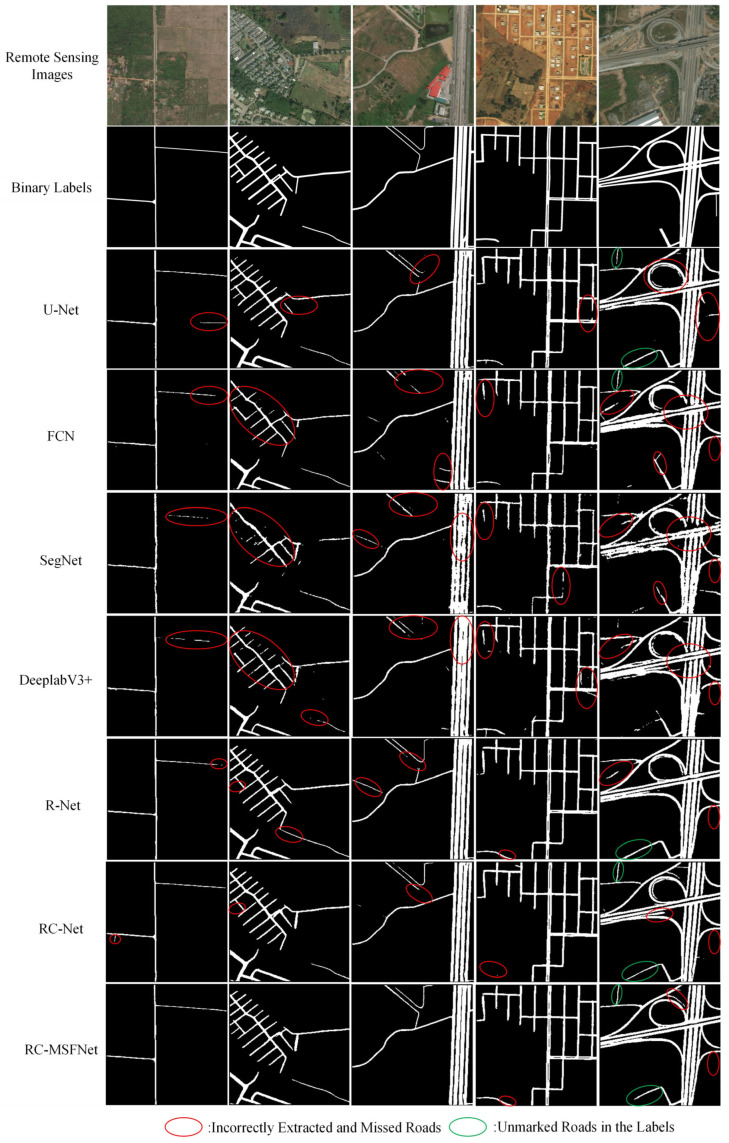
Extraction Results of Roads Using Different Models on the DeepGlobe Dataset.

Figure 11 shows the road extraction results of the DeepGlobe dataset of RC-MSFNet, U-Net, FCN, SegNet, DeeplabV3+, R-Net, and RC-Net models.

As shown in Figure 11, The RC-MSFNet model can also achieve good road extraction results on the DeepGlobe dataset, with fewer errors and omissions compared to U-Net, FCN, SegNet, DeeplabV3+, R-Net, and RC-Net models. It can also extract unlabeled roads from labels. Compared with U-Net, FCN, and SegNet models, the RC-MSFNet model can fully extract main roads and avoid incomplete extraction of main roads. Compared to the DeeplabV3+ model, the RC MSFNet model, with the introduction of the ResNet and CoA modules, can better handle the extraction and recognition of rural roads, preventing the phenomenon of missed extraction of rural roads. Compared to R-Net and RC-Net models, the RC-MSFNet model, with the introduction of the MSF module, better solves the problem of extracting details from narrow roads. The RC-MSFNet model can demonstrate good road extraction performance on the DeepGlobe dataset.

## 5. Conclusions

This study addresses the challenges of incomplete and inaccurate rural road extraction from remote sensing imagery due to narrow and elongated roads, ambiguous boundaries, and textures similar to the surrounding environment. In the RC-MSFNet model, an enhancement of the U-Net model, a ResNet was integrated into the downsampling region to address the vanishing gradient problem. In the bottleneck region, the dual convolution layers were replaced with MSF, allowing the model to adapt to the varying complexities of rural roads. Additionally, CoA was introduced in the upsampling region to enhance the model’s ability to recognize rural roads accurately. Using China’s Xiong’an New Area as the study area and GF-2 satellite remote sensing images as the data source, an XARoads dataset was created through visual interpretation and manual delineation of binary labels on preprocessed images. By optimizing the parameters of the RC-MSFNet network and conducting relevant experiments using the XARoads dataset and DeepGlobe dataset, we aim to explore effective methods for extracting rural roads. The research findings indicate that:

(1) By optimizing the RC-MSFNet model parameters, all road extraction metrics reach their optimal values when the *d* parameters in the MSF module are set to 1, 2, 2, 1, and the *r* parameter in the CoA module is set to 32. This indicates that the RC-MSFNet model is feasible and effective for rural road extraction in high-resolution remote sensing images.
(2) The RC-MSFNet model achieved P, F1, IOU, and COM values of 0.8350, 0.7896, 0.6523, and 0.7489 for rural road extraction in the Xiong’an New Area (XARoads dataset). Compared to models like U-Net, FCN, SegNet, DeeplabV3+, R-Net, and RC-Net, RC-MSFNet demonstrates better performance in extracting narrow rural roads, muddy roads with unclear boundaries, and roads obscured by shadows, with fewer instances of missed or incorrect extractions. Additionally, it can identify roads labeled outside the ground truth.
(3) For the DeepGlobe public dataset, RC-MSFNet achieved P, F1, IOU, and COM values of 0.8266, 0.7821, 0.6380, and 0.7422, further validating the model’s applicability and scalability. This shows that RC-MSFNet is effective for this study’s XARoads dataset and publicly available datasets like DeepGlobe.

This study presents a method that effectively extracts characteristic information about rural roads from high-resolution remote sensing imagery, achieving satisfactory overall extraction results. It provides a viable approach and framework for extracting rural roads from domestically produced high-resolution imagery. Future research will focus on improving the connectivity of rural roads through image post-processing techniques to address issues of road fragmentation caused by occlusions or noise. Additionally, the study will explore strategies based on topological structure optimization to ensure the road network’s overall coherence and topological consistency, enhancing the model’s adaptability in complex environments and its robustness in practical applications.

## Figures and Tables

**Table 1:** Downsampling Block Details.

Downsampling Block	Input	Output	Type	Kernel	Stride	Padding
Encoder1	1024 × 1024 × 3	1024 × 1024 × 64	Double Conv+ResNet	3 × 3	1	1
	1024 × 1024 × 64	512 × 512 × 64	Max pooling	2 × 2	2	0
Encoder2	512 × 512 × 64	512 × 512 × 128	Double Conv+ResNet	3 × 3	1	1
	512 × 512 × 128	256 × 256 × 128	Max pooling	2 × 2	2	0
Encoder3	256 × 256 × 128	256 × 256 × 256	Double Conv+ResNet	3 × 3	1	1
	256 × 256 × 256	128 × 128 × 256	Max pooling	2 × 2	2	0
Encoder4	128 × 128 × 256	128 × 128 × 512	Double Conv+ResNet	3 × 3	1	1
	128 × 128 × 512	64 × 64 × 512	Max pooling	2 × 2	2	0

**Table 2:** MSF Block Details.

MSF Branch	Input	Output	Type	Dilation Rate	Kernel	Stride	Padding
Branch0	64 × 64 × 512	64 × 64 × 512	Dilated Conv	1	3 × 3	1	1
Branch1	64 × 64 × 512	64 × 64 × 512	Dilated Conv	2	3 × 3	1	2
Branch2	64 × 64 × 512	64 × 64 × 512	Dilated Conv	2	3 × 3	1	2
Branch3	64 × 64 × 512	64 × 64 × 512	Dilated Conv	1	3 × 3	1	1
Connect	64 × 64 × 2048	64 × 64 × 1024	Conv	-	1 × 1	1	0

**Table 3:** Upsampling Block Details.

Upsampling Block	Input	Output	Type	Kernel	Stride	Padding
Decoder1	64 × 64 × 1024	128 × 128 × 512	Upsampling	2 × 2	2	0
	128 × 128 × 512	128 × 128 × 1024	Skip connection (Encoder4)	-	-	-
	128 × 128 × 1024	128 × 128 × 512	Double Conv+CoA	3 × 3	1	1
Decoder2	128 × 128 × 512	256 × 256 × 256	Upsampling	2 × 2	2	0
	256 × 256 × 256	256 × 256 × 512	Skip connection (Encoder3)	-	-	-
	256 × 256 × 512	256 × 256 × 256	Double Conv+CoA	3 × 3	1	1
Decoder3	256 × 256 × 256	512 × 512 × 128	Upsampling	2 × 2	2	0
	512 × 512 × 128	512 × 512 × 256	Skip connection (Encoder2)	-	-	-
	512 × 512 × 256	512 × 512 × 128	Double Conv+CoA	3 × 3	1	1
Decoder4	512 × 512 × 128	1024 × 1024 × 64	Upsampling	2 × 2	2	0
	1024 × 1024 × 64	1024 × 1024 × 128	Skip connection (Encoder1)	-	-	-
	1024 × 1024 × 128	1024 × 1024 × 64	Double Conv+CoA	3 × 3	1	1
	1024 × 1024 × 64	1024 × 1024 × 1	Conv	1 × 1	1	0

**Table 4:** Comparative Experimental Model.

No.	Model	Description
1	U-Net	Encoder–decoder architecture
2	FCN	Fully Convolutional Network
3	SegNet	Encoder–decoder architecture
4	Deeplab V3+	Dilated convolution and encoder–decoder architecture
5	R-Net	U-Net with ResNet added to the downsampling
6	RC-Net	U-Net with ResNet added to the downsampling and CoA to the upsampling

**Table 5:** RC-MSFNet Model’s Different Parameter Rural Road Extraction Results.

No.	Parameter Settings		P	F1	IOU	COM
*A*	MSF (*d* ^1^ = 1.2.4.8)	CoA (*r* ^2^ = 4)	**0.8464** ^3^	0.7801	0.6395	0.7234
	MSF (*d* = 1.2.4.8)	CoA (*r* = 8)	0.8462	0.7799	0.6392	0.7233
	MSF (*d* = 1.2.4.8)	CoA (*r* = 16)	0.8247	0.7807	0.6403	0.7412
	MSF (*d* = 1.2.4.8)	CoA (*r* = 32)	0.8193	0.7699	0.6258	0.7261
*B*	MSF (*d* = 1.4.8.12)	CoA (*r* = 4)	0.8212	0.7771	0.6354	0.7374
	MSF (*d* = 1.4.8.12)	CoA (*r* = 8)	0.8336	0.7670	0.6220	0.7102
	MSF (*d* = 1.4.8.12)	CoA (*r* = 16)	0.8311	0.7712	0.6276	0.7193
	MSF (*d* = 1.4.8.12)	CoA (*r* = 32)	0.8119	0.7820	0.6421	0.7442
*C*	MSF (*d* = 1.2.2.1)	CoA (*r* = 4)	0.8263	0.7710	0.6273	0.7227
	MSF (*d* = 1.2.2.1)	CoA (*r* = 8)	0.8458	0.7800	0.6393	0.7177
	MSF (*d* = 1.2.2.1)	CoA (*r* = 16)	0.8214	0.7662	0.6210	0.7179
	MSF (*d* = 1.2.2.1)	CoA (*r* = 32)	0.8350	**0.7896**	**0.6523**	**0.7489**

^1^ Refers to the dilation rates at different scales in MSF. ^2^ Denotes the reduction factor for the number of channels in CoA. ^3^ Bold text indicates optimal values for each column.

**Table 6:** Different Models’ Rural Road Extraction Results on the XARoads Dataset.

Model	P	F1	IOU	COM
U-Net	0.7970	0.7414	0.5890	0.6930
FCN	0.7672	0.6833	0.5190	0.6160
SegNet	0.7565	0.6820	0.5174	0.6209
DeeplabV3+	0.8136	0.6981	0.5362	0.6114
R-Net	0.8292	0.7592	0.6119	0.7001
RC-Net	0.8097	0.7645	0.6188	0.7241
RC-MSFNet	**0.8350** ^1^	**0.7896**	**0.6523**	**0.7489**

^1^ Bold text indicates the optimal values for each column.

**Table 7:** Different Models’ Rural Road Extraction Results on the DeepGlobe Dataset.

Model	P	F1	IOU	COM
U-Net	0.7979	0.7658	0.6204	0.7361
FCN	0.7829	0.6787	0.5137	0.6126
SegNet	0.7663	0.6383	0.4688	0.5469
DeeplabV3+	0.7945	0.6912	0.5281	0.6116
R-Net	0.8118	0.7663	0.6211	0.7256
RC-Net	0.8186	0.7769	0.6352	0.7393
**RC-MSFNet**	**0.8266** ^1^	**0.7821**	**0.6380**	**0.7422**

^1^ Bold text indicates the optimal values for each column.

## Data Availability

The DeepGlobe Dataset is publicly accessible at https://www.kaggle.com/ (accessed on 14 October 2024). For the private XARoads datasets, requests for access can be directed to yangnj2022@163.com.

## References

[1] Li Z., Zhang S., Dong J. (2022). Suggestive data annotation for CNN-based building footprint mapping based on deep active learning and landscape metrics. Remote Sens..

[2] Alshehhi R., Marpu P.R. (2017). Hierarchical graph-based segmentation for extracting road networks from high-resolution satellite images. ISPRS J. Photogramm. Remote Sens..

[3] Alshehhi R., Marpu P.R., Woon W.L., Dalla Mura M. (2017). Simultaneous extraction of roads and buildings in remote sensing imagery with convolutional neural networks. ISPRS J. Photogramm. Remote Sens..

[4] Unsalan C., Sirmacek B. (2012). Road network detection using probabilistic and graph theoretical methods. IEEE Trans. Geosci. Remote Sens..

[5] Fang Y.P., Wang X.P., Li X.N. (2022). Road Extraction from Remote Sensing Images Based on Adaptive Morphology. Laser Optoelectron. Prog..

[6] Feng H.X., Li J.Y., Wang Y.M. (2021). Research on Road Extraction from Remote Sensing Image Based on Improved Support Vector Machine. J. Zhejiang Univ. Water Resour. Electr. Power.

[7] Zhu D.M., Wen X., Ling C.L. Road extraction based on the algorithms of MRF and hybrid model of SVM and FCM. Proceedings of the 2011 International Symposium on Image and Data Fusion.

[8] Sharma P., Kumar R., Gupta M. Road Features Extraction Using Convolutional Neural Network. Proceedings of the 2023 International Conference on Advancement in Computation & Computer Technologies (InCACCT).

[9] Sharma P., Kumar R., Gupta M., Nayyar A. (2024). A critical analysis of road network extraction using remote sensing images with deep learning. Spat. Inf. Res..

[10] Papadomanolaki M., Vakalopoulou M., Karantzalos K. Patch-based deep learning architectures for sparse annotated very high resolution datasets. Proceedings of the 2017 Joint Urban Remote Sensing Event (JURSE).

[11] Cheng G., Wang Y., Xu S., Wang H., Xiang S., Pan C. (2017). Automatic road detection and centerline extraction via cascaded end-to-end convolutional neural network. IEEE Trans. Geosci. Remote Sens..

[12] Zhang Z., Liu Q., Wang Y. (2018). Road extraction by deep residual u-net. IEEE Geosci. Remote Sens. Lett..

[13] Xu Y., Feng Y., Xie Z., Hu A., Zhang X. A research on extracting road network from high resolution remote sensing imagery. Proceedings of the 2018 26th International Conference on Geoinformatics.

[14] Buslaev A., Seferbekov S., Iglovikov V., Shvets A. Fully convolutional network for automatic road extraction from satellite imagery. Proceedings of the IEEE Conference on Computer Vision and Pattern Recognition Workshops.

[15] Yang X., Li X., Ye Y., Zhang X., Zhang H., Huang X., Zhang B. Road detection via deep residual dense u-net. Proceedings of the 2019 International Joint Conference on Neural Networks (IJCNN).

[16] Chen L.C., Papandreou G., Kokkinos I., Murphy K., Yuille A.L. (2016). Semantic Image Segmentation with Deep Convolutional Nets and Fully Connected CRFs. arXiv.

[17] Chen L.C., Zhu Y., Papandreou G., Schroff F., Adam H. Encoder-decoder with atrous separable convolution for semantic image segmentation. Proceedings of the European Conference on Computer Vision (ECCV).

[18] Xia W., Zhang Y.Z., Liu J., Luo L., Yang K. (2018). Road extraction from high resolution image with deep convolution network—A case study of GF-2 image. Proceedings.

[19] Li H., Chao Y., Yu P., Li H., Zhang Y. (2023). Image Inpainting Algorithm with Diverse Aggregation of Contextual Information. J. Beijing Univ. Posts Telecommun..

[20] Wang Q., Bai H., He C., Cheng J. FE-LinkNet: Enhanced D-LinkNet with Attention and Dense Connection for Road Extraction in High-Resolution Remote Sensing Images. Proceedings of the IGARSS 2022—2022 IEEE International Geoscience and Remote Sensing Symposium.

[21] Demir I., Koperski K., Lindenbaum D., Pang G., Huang J., Basu S., Raskar R. Deepglobe 2018: A challenge to parse the earth through satellite images. Proceedings of the IEEE Conference on Computer Vision and Pattern Recognition Workshops.

[22] Doshi J. Residual inception skip network for binary segmentation. Proceedings of the IEEE Conference on Computer Vision and Pattern Recognition Workshops.

[23] He K., Zhang X., Ren S., Sun J. Deep residual learning for image recognition. Proceedings of the IEEE Conference on Computer Vision and Pattern Recognition.

[24] Yu F. (2015). Multi-scale context aggregation by dilated convolutions. arXiv.

[25] Chen D., Li X., Hu F., Mathiopoulos P.T., Di S., Sui M., Peethambaran J. (2023). Edpnet: An encoding–decoding network with pyramidal representation for semantic image segmentation. Sensors.

[26] Zhou L., Zhang C., Wu M. D-LinkNet: LinkNet with pretrained encoder and dilated convolution for high resolution satellite imagery road extraction. Proceedings of the IEEE Conference on Computer Vision and Pattern Recognition Workshops.

[27] Guo M., Liu H., Xu Y., Huang Y. (2020). Building extraction based on U-Net with an attention block and multiple losses. Remote Sens..

[28] Vaswani A. (2017). Attention is all you need. Advances in Neural Information Processing Systems.

[29] Sanghyun W., Jongchan P., Joon-Young L., In S.K. CBAM: Convolutional block attention module. Proceedings of the European Conference on Computer Vision (ECCV).

[30] Li P., He X., Qiao M., Cheng X., Li Z., Luo H., Tian Z. (2020). Robust deep neural networks for road extraction from remote sensing images. IEEE Trans. Geosci. Remote Sens..

[31] Wang Y., Zeng X.Q. (2022). Road extraction model derived from integrated attention mechanism and dilated convolution. J. Image Graph..

[32] Yerram V., Takeshita H., Iwahori Y., Hayashi Y., Bhuyan M.K., Fukui S., Wang A. (2022). Extraction and calculation of roadway area from satellite images using improved deep learning model and post-processing. J. Imaging.

